# Decoding the heterogeneity of liver-resident macrophages in chronic liver diseases: therapeutic responses to immunomodulatory strategies

**DOI:** 10.3389/fphar.2025.1708240

**Published:** 2025-11-10

**Authors:** Renbin Ouyang, Xiaocheng Li, Jianhua Hao, Jie Lin, Hui Lan, Jing Peng, Xinmin Li, Zhiliang Tian, Yu Sun

**Affiliations:** 1 Department of Hepatobiliary Surgery, Loudi Central Hospital, Loudi, Hunan, China; 2 Department of Hepatobiliary Surgery, The First Affiliated Hospital of Hunan University of Medicine, Huaihua, Hunan, China; 3 Department of Hepatobiliary Surgery, The Second Affiliated Hospital of Chongqing Medical University, Chongqing, China; 4 Department of Clinical Nutrition, The Second Affiliated Hospital of Chongqing Medical University, Chongqing, China

**Keywords:** liver-resident macrophage, Kupffer cell, monocyte-derived macrophage, chronic liver disease, immunomodulatory therapy, heterogeneity

## Abstract

Chronic liver diseases (CLDs), encompassing a spectrum of etiologies including metabolic dysfunction, alcohol abuse, and viral infections, represent a significant global health burden. The progression of these diseases to fibrosis, cirrhosis, and hepatocellular carcinoma is underpinned by complex immunological mechanisms in which liver-resident macrophages (LRMs) are central players. LRMs are not a monolithic population but a heterogeneous consortium of cells, primarily comprising embryonically-derived, self-renewing Kupffer cells and dynamically recruited monocyte-derived macrophages. These subsets, along with newly identified populations like lipid-associated macrophages and scar-associated macrophages, exhibit distinct origins, phenotypes, and functions that profoundly influence the trajectory of liver injury and repair. A new generation of immunomodulatory therapies is being developed to specifically target the pathways that govern LRM function. However, clinical responses to these agents have been variable, a phenomenon largely attributable to their differential effects on the diverse LRM subsets and the profound heterogeneity of the patient population. This review elucidates the complex heterogeneity of LRMs in the context of different CLDs. We dissect the mechanisms by which emerging immunomodulatory therapies—including PPAR agonists, chemokine receptor antagonists, and intracellular signaling inhibitors—alter the balance, phenotype, and functional output of distinct LRM populations. By integrating findings from preclinical models with outcomes from recent clinical trials, we illustrate how the specific modulation of LRM subsets correlates with therapeutic efficacy or failure. Furthermore, we discuss the critical role of LRMs in the progression to hepatocellular carcinoma and the implications for immune checkpoint inhibitor therapies. Finally, we outline the key challenges in translating these findings into clinical practice and highlight future research priorities, emphasizing the need for single-cell technologies, investigation of the gut-liver axis, and development of combination therapies. A deeper understanding of LRM biology is paramount to advancing a precision medicine approach, ultimately paving the way for more effective and personalized treatments for patients with CLD.

## 1 Introduction

The therapeutic paradigm for chronic liver diseases (CLDs), such as metabolic dysfunction-associated steatohepatitis (MASH), alcohol-associated liver disease (ALD), and viral hepatitis, is undergoing a profound transformation. Historically, treatment was largely confined to managing primary causes or addressing end-stage complications. The contemporary approach, however, is guided by a sophisticated understanding of the immunological drivers of liver inflammation, fibrogenesis, and oncogenesis, leading to the development of targeted immunomodulatory therapies (Kamata et al., 2023; Hu et al., 2024; Gilgenkrantz et al., 2025). These novel agents are designed to precisely manipulate specific immune cell populations and signaling pathways, moving beyond the limitations of broad-spectrum immunosuppression. The therapeutic arsenal now includes a diverse array of agents, from monoclonal antibodies targeting immune checkpoints like programmed cell death-1/programmed death-ligand 1 (PD-1/PD-L1) to small molecule inhibitors that modulate key intracellular signaling cascades (Mohr et al., 2021; Gao et al., 2022; Nathani and Bansal, 2023). However, the clinical application of these therapies has yielded heterogeneous outcomes, with some patients experiencing significant benefits while others show minimal response or disease progression. This variability highlights that CLD pathogenesis is not a monolithic process but rather a consequence of the intricate crosstalk among diverse cell types within the liver microenvironment (Saldarriaga et al., 2023).

Central to this intricate network are the liver-resident macrophages (LRMs), the most abundant immune cell population in the liver. Strategically positioned within the hepatic sinusoids, they act as indispensable sentinels and master regulators of homeostasis, inflammation, and repair (van der Heide et al., 2019). In health, LRMs maintain an immunologically tolerant state, crucial for processing gut-derived antigens without inciting undue inflammation (Ma et al., 2024). They are vital for clearing pathogens, cellular debris, and metabolic byproducts. With the onset of chronic injury, their role shifts dramatically from guardians of homeostasis to drivers of pathology (Elsherif and Alm, 2022; Guan et al., 2025). Activated by damage-associated molecular patterns (DAMPs) and pathogen-associated molecular patterns (PAMPs), LRMs release a torrent of pro-inflammatory and pro-fibrotic mediators (Ma et al., 2024). This persistent activation establishes a pathogenic feedback loop involving hepatocyte death, inflammation, and the stimulation of hepatic stellate cells (HSCs), which in turn drives the progressive deposition of extracellular matrix (ECM) characteristic of fibrosis (Guo Z. et al., 2024; Ran et al., 2025).

Crucially, the term “LRM” encompasses a functionally and ontogenically diverse population. The two principal subsets are the embryonically derived, self-renewing Kupffer cells (KCs) and the dynamically recruited, bone marrow-derived monocyte-derived macrophages (MoMφs) (van der Heide et al., 2019; Flores Molina et al., 2022). KCs are the long-term residents, integral to the liver’s architecture and homeostatic functions (Elsherif and Alm, 2022; Flores Molina et al., 2022; Bennett et al., 2023). In contrast, MoMφs are rapidly recruited from the circulation during injury, primarily via the CCL2-CCR2 chemokine axis (Ma et al., 2024; Qian et al., 2024). Once in the liver, they exhibit remarkable plasticity, differentiating into functionally distinct subsets, classically distinguished in murine models by Ly-6C expression. Functionally, the Ly-6C^hi^ subset predominantly executes pro-inflammatory and pro-fibrotic programs, while the Ly-6C^lo^ subset is chiefly implicated in tissue repair and the resolution of fibrosis (Elsherif and Alm, 2022; Ma et al., 2024; Ran et al., 2025). This functional dichotomy between KCs and MoMφs, and among MoMφ subsets, is a cornerstone of CLD pathogenesis and a critical consideration for therapeutic design (Bennett et al., 2023).

**FIGURE 1:**
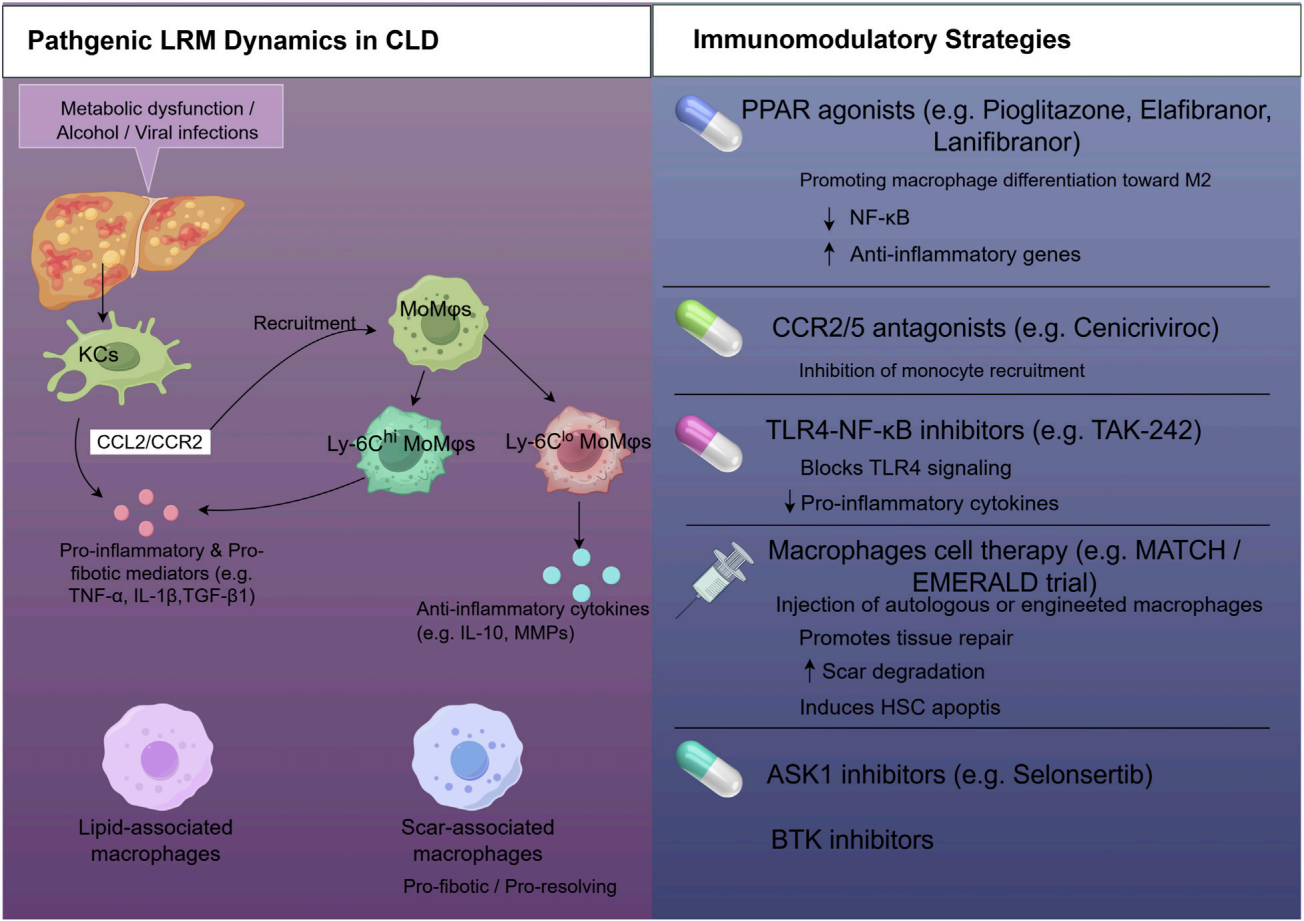
Therapeutic targeting of liver-resident macrophages (LRMs) in chronic liver diseases (CLD). This schematic illustrates the pathogenic dynamics of LRMs in CLD and outlines key immunomodulatory strategies. Left panel: Initial insults like metabolic stress or viral infection activate Kupffer cells (KCs), making them pro-inflammatory and pro-fibrotic. Activated KCs (potentially expressing CCL2/CCR2) recruit monocyte-derived macrophages (MoMφs) that also adopt pro-inflammatory and pro-fibrotic phenotypes. Specialized LRM subsets, such as lipid-associated macrophages (LAMs) and scar-associated macrophages (SAMs), emerge in specific pathological contexts, contributing to liver injury and fibrotic scar formation. Right panel: Immunomodulatory strategies. The ultimate objective of these strategies is to modulate LRM subsets to achieve fibrosis resolution and improve liver outcomes. Abbreviations: ASK1, apoptosis signal-regulating kinase 1; BTK, Bruton’s tyrosine kinase; CCL2/CCR2, chemokine ligand 2/chemokine receptor 2; MMPs, matrix metalloproteinases; PRRs, pattern recognition receptors; TLRs, toll-like receptors.

The central thesis of this review is that the clinical efficacy of immunomodulatory agents in CLDs is fundamentally dependent on their differential impact on these heterogeneous LRM populations. Consequently, a “one-size-fits-all” approach for macrophage-targeted therapy is unlikely to succeed given the distinct biology of KCs versus MoMφs. A comprehensive understanding of how specific therapies reshape the LRM landscape is essential for advancing precision medicine in hepatology. This review will systematically deconstruct the mechanisms by which these interventions alter the abundance, phenotype, and function of different LRM subsets. By integrating preclinical and clinical evidence, we will explore how these cellular shifts correlate with disease outcomes. Ultimately, this analysis aims to provide a framework for developing next-generation therapeutic strategies that can selectively target pathogenic macrophage subsets while preserving or enhancing reparative ones, thereby heralding a new era of personalized treatment for CLDs. Figure 1 provides a comprehensive schematic overview of LRM heterogeneity in health and disease, highlighting the pathogenic dynamics of specific LRM subsets and key immunomodulatory strategies aimed at their therapeutic targeting.

## 2 LRM subsets in health and disease

### 2.1 KCs

KCs are the archetypal tissue-resident macrophages of the liver, defined by their unique embryonic provenance and their capacity for self-renewal (Flores Molina et al., 2022). Originating from yolk sac and fetal liver progenitors during development, KCs establish a stable, long-lived population that maintains itself through *in situ* proliferation, independent of replenishment from circulating bone marrow-derived monocytes (Tacke, 2017; Bennett et al., 2023). This autonomy allows KCs to become deeply integrated into the liver’s microarchitecture, forming a lasting cellular network that contributes to their specialized roles in immune surveillance and metabolic homeostasis. This developmental origin confers lasting functional consequences, with certain core homeostatic roles being exclusively executed by this authentic KC population and not fully recapitulated by monocyte-derived cells that engraft the liver following KC depletion (Blériot and Ginhoux, 2019). Furthermore, the stable, self-renewing nature of KCs means that epigenetic modifications or functional adaptations acquired in response to chronic stimuli, such as a high-fat diet or alcohol, can be perpetuated within the KC pool, establishing a form of pathological memory that may contribute to a persistent pro-inflammatory state even after the initial insult is removed (Musrati et al., 2024).

By virtue of their strategic positioning within the hepatic sinusoids, KCs form the liver’s primary line of defense, filtering blood arriving directly from the gastrointestinal tract (Lopes et al., 2022; Ma et al., 2024). Equipped with a broad array of pattern recognition receptors (PRRs) like Toll-like receptors (TLRs), they detect microbial products (PAMPs) and endogenous danger signals (DAMPs), initiating rapid innate immune responses to control infection (Zhou et al., 2022; Ma et al., 2023). Beyond their defensive duties, KCs are critical for maintaining tissue homeostasis. As highly efficient phagocytes, they clear apoptotic cells, cellular debris, and circulating immune complexes, a process that is actively immunoregulatory and prevents autoimmune reactions (Guan et al., 2025). KCs also contribute directly to tissue regeneration. Following acute liver injury, they secrete growth factors, such as hepatocyte growth factor (HGF), which stimulate hepatocyte proliferation and drive the restoration of liver mass and function (Lkham-Erdene et al., 2024; Guan et al., 2025; Zhao et al., 2025). This highlights the dual capacity of KCs to both initiate inflammation and orchestrate its resolution and subsequent repair.

In the setting of CLD, the homeostatic functions of KCs are subverted (Wen et al., 2021; Yang et al., 2023). Persistent exposure to injurious stimuli—lipotoxic metabolites in MASH, ethanol and its byproducts in ALD, or viral components—triggers chronic KC activation (Ma et al., 2024). This shift transforms them into pro-inflammatory and pro-fibrotic effectors. Activated KCs are characterized by a pro-inflammatory secretome, releasing key mediators such as TNF-α, IL-1β, and chemokines like CCL2, which perpetuate hepatocyte injury, recruit additional inflammatory cells, and directly activate HSCs to produce collagen (Gao et al., 2022; Seo et al., 2022; 2023; Jung et al., 2024). Over time, this chronic insult can lead to KC senescence, characterized by a pro-inflammatory secretome that further fuels the disease process (Bai et al., 2025). In severe injury, the embryonic KC population can become depleted and replaced by monocyte-derived cells (Wang et al., 2021; Hirako et al., 2022). These monocyte-derived replacements may not fully replicate the homeostatic functions of their predecessors, creating a long-term deficit in hepatic immune regulation that can exacerbate ongoing tissue damage (Parthasarathy and Malhi, 2021; Su et al., 2021; Wang et al., 2021; Guo W. et al., 2024).

### 2.2 MoMφs

Unlike the stable KC population, a substantial influx of MoMφs is rapidly recruited from the bone marrow to the liver in response to injury. This recruitment is a defining feature of hepatic inflammation and is governed primarily by the CCL2-CCR2 chemokine axis (Ruiqi et al., 2023; Qian et al., 2024). Damaged hepatocytes and activated KCs release CCL2, creating a potent chemoattractant gradient that guides the migration of circulating CCR2-expressing monocytes into the liver sinusoids (Wen et al., 2021; Lee et al., 2022; Sauer et al., 2024). These cells then transmigrate into the liver parenchyma, where they differentiate into mature macrophages. The critical role of this pathway is demonstrated by preclinical studies where genetic or pharmacological blockade of CCR2 significantly reduces macrophage infiltration and ameliorates liver injury and fibrosis (Guillot and Tacke, 2023; Nathani and Bansal, 2023). Whereas this process is essential for resolving acute insults, its unabated persistence in chronic disease states transforms MoMφs into key drivers of pathology (Kohlhepp et al., 2023; Sanchez Vasquez et al., 2025).

Upon entering the hepatic parenchyma, MoMφs exhibit remarkable functional polarization, differentiating into functionally distinct subsets (Sayaf et al., 2023). In murine models, these are often categorized based on the expression of the surface marker Ly-6C (Sayaf et al., 2023; Ma et al., 2024). The Ly-6C^hi^ subset represents the classically activated, pro-inflammatory macrophages. These cells constitute the initial wave of infiltrating responders and are potent producers of inflammatory cytokines (TNF-α, IL-1β) and reactive oxygen species (ROS) (Tada et al., 2022; Makiuchi et al., 2023; Ma et al., 2024). In chronic disease, their sustained presence perpetuates inflammation and they serve as a major source of pro-fibrotic mediators like TGF-β1, which directly activate HSCs (Li Y.-H. et al., 2021; Pan et al., 2024).

In contrast, the Ly-6C^lo^ subset embodies an anti-inflammatory and pro-resolving phenotype (Sayaf et al., 2023). These macrophages are crucial for orchestrating tissue repair and the resolution of fibrosis. They are characterized by the production of anti-inflammatory cytokines like IL-10 and, critically, matrix metalloproteinases (MMPs), such as MMP13, which are enzymes that degrade the fibrotic scar (Fallowfield et al., 2007). The dynamic transition from a pro-inflammatory Ly-6C^hi^ state to a reparative Ly-6C^lo^ state is a pivotal event in the healing process. This phenotypic switch represents a critical checkpoint that determines whether liver injury progresses to fibrosis or resolves.

This functional dichotomy illustrates that MoMφs span the full spectrum of the liver’s response to injury. In the initial phase, the influx of Ly-6C^hi^ MoMφs amplifies inflammation and initiates fibrogenesis by activating HSCs. This sustained influx is a key feature of progressive CLDs (Riad et al., 2023). However, these same cells are also indispensable for healing. Upon removal of the injurious stimulus or as the local microenvironment changes, MoMφs can switch to a reparative Ly-6C^lo^ phenotype. These reparative macrophages actively dismantle the fibrotic scar by producing MMPs and promote the clearance of activated HSCs through apoptosis. This dual role makes MoMφs a compelling therapeutic target. Consequently, the therapeutic goal is not simple ablation, but rather a nuanced modulation of their function: to block the recruitment of pathogenic Ly-6C^hi^ cells while simultaneously promoting the emergence and pro-resolving functions of the Ly-6C^lo^ subset, thereby tipping the balance from fibrogenesis towards resolution.

### 2.3 LAMs and SAMs

The advent of high-resolution technologies, chief among them single-cell RNA sequencing (scRNA-seq), has fundamentally reshaped the conceptual framework of macrophage heterogeneity beyond previous classifications, leading to the identification of novel subsets linked to specific pathologies (Wang et al., 2023; Li B. et al., 2024). In the context of MASH, a distinct population known as lipid-associated macrophages (LAMs) has been identified (Shi S. et al., 2025). These cells are defined by both their spatial proximity to lipid-laden hepatocytes and a distinctive transcriptional program, which includes high expression of the triggering receptor expressed on myeloid cells 2 (TREM2) (Ganguly et al., 2024; Xu et al., 2025). The abundance of TREM2+ LAMs strongly correlates with disease severity in both human MASH and corresponding mouse models (Ganguly et al., 2024; Shi S. et al., 2025). Analogously, another specialized subset, designated scar-associated macrophages (SAMs), has been described within the fibrotic niche of the liver (Fallowfield et al., 2007; Fabre et al., 2023). SAMs, which can arise from both KCs and MoMφs, are characterized by a gene expression profile that reflects their integral role in tissue remodeling and fibrogenesis. The discovery of these context-dependent macrophage populations illustrates a core principle: the local tissue microenvironment actively shapes macrophage identity and function.

LAMs and SAMs play distinct roles directly related to the pathological hallmarks of MASH and fibrosis. The functional role of TREM2+ LAMs appears to be multifaceted and context-dependent (Ganguly et al., 2024). While their presence is associated with disease severity, TREM2 is involved in lipid clearance and phagocytosis of apoptotic cells, suggesting a potentially protective role aimed at removing damaged, lipotoxic hepatocytes (Shi S. et al., 2025). Indeed, studies in TREM2-deficient mice have shown exacerbated MASH, supporting a net protective function for these cells (Ganguly et al., 2024). This suggests a model wherein LAMs initially serve a protective function, but that their reparative capacity becomes overwhelmed or corrupted by the persistently lipotoxic environment, leading to maladaptive outcomes.

SAMs, topographically situated within the dense fibrotic scar, are pivotal regulators of ECM dynamics (Fallowfield et al., 2007; Fabre et al., 2023). They are a major source of pro-fibrotic mediators that sustain HSC activation. However, reflecting macrophage plasticity, SAMs also possess the capacity for resolution. They are a critical source of MMP13, the primary collagenase responsible for degrading the fibrotic scar during the resolution phase (Fallowfield et al., 2007). Therefore, the functional balance of SAMs acts as a fulcrum, determining whether the fibrotic scar undergoes progressive accumulation or resolution. Targeted reprogramming of these specialized macrophage subsets—shifting them from a pathogenic to a reparative state—thus represents a promising therapeutic frontier for treating advanced liver disease.

## 3 Therapeutic interventions targeting LRMs

### 3.1 Modulating macrophage polarization: the M1/M2 axis

Peroxisome proliferator-activated receptors (PPARs) are nuclear hormone receptors that function as pivotal transcriptional regulators of metabolism and inflammation (Kazankov et al., 2019; Feng et al., 2021). Agonists of these receptors have emerged as leading therapeutic candidates for MASH, principally through their capacity to modulate macrophage polarization. PPAR agonists, particularly those targeting PPAR-γ and PPAR-δ, directly influence macrophage function by skewing macrophage polarization from a pro-inflammatory M1 phenotype toward an anti-inflammatory, tissue-reparative M2 phenotype (Luo et al., 2017; Kazankov et al., 2019; Yang et al., 2025). This is mechanistically accomplished by inhibiting the activity of pro-inflammatory transcription factors like NF-κB while simultaneously upregulating genes characteristic of M2 macrophages, such as the mannose receptor (MR) and arginase 1 (Arg1). This reprogramming of KCs and other LRMs is crucial for attenuating the chronic inflammation that drives MASH progression (Ma et al., 2024).

The clinical development of PPAR agonists has yielded mixed but informative results. Pioglitazone (PPAR-γ) has demonstrated efficacy in improving steatosis and inflammation, though its impact on fibrosis is less consistent (Jacques et al., 2021; Cho et al., 2023; Mazhar et al., 2023; Inia et al., 2025). Elafibranor (PPAR-α/δ) showed promise in a Phase II trial but failed to meet its primary endpoint in the subsequent Phase III RESOLVE-IT study (Kazankov et al., 2019; Cho et al., 2023; Levy et al., 2025). The most promising results to date have come from the pan-PPAR agonist lanifibranor, which targets all three isoforms (α, δ, and γ). In the Phase IIb NATIVE trial, lanifibranor met both primary endpoints relevant for accelerated approval: NASH resolution without worsening of fibrosis and fibrosis improvement without worsening of NASH (Sven et al., 2020; Francque et al., 2021). These results, attributed to its comprehensive targeting of metabolic, inflammatory, and fibrotic pathways, have propelled lanifibranor into Phase III trials (Francque et al., 2021).

The TLR4-NF-κB signaling axis represents a canonical pathway in innate immunity and a key driver that induces M1 macrophage polarization in CLDs (Tang et al., 2023; Pu et al., 2025). In ALD, gut-derived lipopolysaccharide (LPS) activates TLR4 on KCs, while in MASH, endogenous ligands released from damaged cells serve as activators. This engagement initiates a downstream signaling cascade culminating in the nuclear translocation of NF-κB and its orchestration of a broad pro-inflammatory gene expression program (Yin et al., 2021; Tang et al., 2023). Inhibitors of this pathway, such as the small molecule TAK-242, block TLR4 signaling, thereby preventing NF-κB activation and the subsequent production of M1-associated cytokines by macrophages. This effectively suppresses the pro-inflammatory state that contributes to hepatocyte damage and fibrosis (Xie et al., 2022). However, the therapeutic implications of targeting this pathway are complex. While its inhibition is beneficial in attenuating the initial inflammatory and fibrogenic response (Ren et al., 2024), the TLR4-NF-κB axis is also implicated in later stages of tissue repair and regeneration (Li S. et al., 2021). Consequently, complete blockade might compromise the reparative phase of healing, as fibrosis resolution also depends on a degree of macrophage activation. This duality highlights the need for a nuanced therapeutic approach that modulates, rather than completely abrogates, this critical signaling pathway.

An innovative strategy involves the direct administration of macrophages polarized *ex vivo* to a desired phenotype. This approach seeks to directly bolster the liver’s reparative capacity (Pouyanfard et al., 2021). Preclinical studies have explored the infusion of bone marrow-derived macrophages polarized to either M1 or M2 states into mice with liver fibrosis (Ma et al., 2017). Counterintuitively, M1-polarized macrophages demonstrated superior therapeutic efficacy. The therapeutic benefit, however, was found to be indirect; they acted as potent modulators of the host immune environment, enhancing the recruitment of endogenous restorative Ly-6C^lo^ macrophages, which then produced MMPs to degrade scar tissue (Ma et al., 2017). The M1 macrophages also boosted the activation of natural killer (NK) cells, which induced apoptosis in activated HSCs (Ma et al., 2017). In contrast, M2-polarized macrophages showed limited efficacy in these models, challenging the simplistic notion that an anti-inflammatory phenotype is always beneficial for resolving established fibrosis.

This preclinical concept is now being translated into the clinic. The MATCH Phase II trial investigated the infusion of autologous, non-engineered macrophages in patients with cirrhosis (Moroni et al., 2019; Brennan et al., 2025). The results showed the treatment was well-tolerated and associated with a significant reduction in clinical complications compared to standard care (Brennan et al., 2025). Building on this, the upcoming EMERALD trial will test “supercharged” macrophages, engineered *ex vivo* to enhance their reparative properties, in patients with advanced cirrhosis, representing a next-generation cell therapy approach.

### 3.2 Blocking macrophage recruitment and trafficking

A prominent therapeutic strategy is to inhibit the recruitment of pro-inflammatory monocytes by targeting the chemokine receptors CCR2 and CCR5, which are pivotal for their migration to the site of hepatic injury (Ruiqi et al., 2023). This strategy is exemplified by cenicriviroc, a dual antagonist of CCR2 and CCR5 (Madan et al., 2024). By blocking these receptors, cenicriviroc prevents the migration of pro-inflammatory Ly-6C^hi^ monocytes into the liver, thereby curtailing the accumulation of pathogenic macrophages that drive inflammation and fibrosis (Wang et al., 2021). A key theoretical advantage of this approach is its selectivity, aiming to disrupt the detrimental influx of MoMφs without perturbing the homeostatic functions of the resident KC population.

The initial promise of this strategy was supported by the Phase IIb CENTAUR trial, where cenicriviroc demonstrated significant anti-fibrotic effects, warranting its progression into a large Phase III program (Francque et al., 2024). However, this initial optimism was tempered when the subsequent Phase III AURORA study failed to meet its primary endpoint, demonstrating no significant benefit of cenicriviroc over placebo for fibrosis improvement in adults with MASH (Anstee et al., 2024). This outcome, despite a robust preclinical and Phase II rationale, serves as a cautionary example, highlighting the challenges of targeting a single pathway in a multifactorial disease and underscoring the confounding effect of the significant placebo response rates frequently observed in MASH trials. Furthermore, this failure likely reflects the profound heterogeneity of the patient population. In patients with established fibrosis, the liver’s pathogenic microenvironment may become self-sustaining, driven by crosstalk between existing LRM populations (like SAMs) and HSCs, thus becoming less reliant on the continuous recruitment of new monocytes. In this context, blocking monocyte influx alone may be insufficient to reverse entrenched fibrosis. Additionally, factors such as genetic background (e.g., PNPLA3 variants), co-existing type 2 diabetes, and variations in the gut microbiome can create distinct disease endotypes where the CCL2-CCR2 axis may not be the dominant pathogenic driver, rendering its blockade insufficient in a broad, unselected patient cohort (Xia et al., 2024; Lindén et al., 2025; Park et al., 2025).

An alternative therapeutic modality involves neutralizing the chemokine ligands directly, rather than blocking their receptors. Pharmacological neutralization of CCL2, for instance with specific inhibitors like RNA aptamers or antibodies, aims to dismantle the chemotactic gradient required for monocyte recruitment (Kang et al., 2023). In preclinical models, this strategy has been shown to effectively curb the infiltration of Ly-6C^hi^ MoMφs, resulting in diminished HSC activation and a consequent reduction in liver fibrosis (Xi et al., 2021). However, despite providing an upstream and targeted intervention, a critical limitation of this strategy is the inherent redundancy of the chemokine network. Targeting a single ligand may be compensated by upregulation of other chemokines (Diao et al., 2025; Geervliet et al., 2025).

### 3.3 Targeting intracellular signaling pathways

Apoptosis signal-regulating kinase 1 (ASK1) functions as a critical signaling hub that integrates cellular stress signals, such as those from oxidative stress, to drive apoptosis and inflammation in both hepatocytes and macrophages (Chen et al., 2023; Thakur et al., 2025). The therapeutic rationale for inhibiting ASK1 with selonsertib was therefore twofold: to concurrently shield hepatocytes from cell death while also attenuating the activation of pro-inflammatory signaling cascades (p38/JNK) within macrophages, thereby decreasing the production of inflammatory cytokines (van der Heide et al., 2019; Hou et al., 2021; Han and Im, 2024). Despite this rationale and promising Phase II data, selonsertib conclusively failed to meet its primary anti-fibrotic endpoints in two large Phase III trials—STELLAR-3 (F3 fibrosis) and STELLAR-4 (F4 cirrhosis)—showing no significant improvement in fibrosis compared to placebo in patients with advanced MASH (Harrison et al., 2020; Rinella and Noureddin, 2020; Loomba et al., 2021). This outcome provides a critical lesson, suggesting that targeting the ASK1 pathway in isolation is likely insufficient to meaningfully alter the trajectory of established, advanced fibrosis. This insufficiency is exacerbated in heterogeneous patient populations where the contribution of ASK1-mediated stress may vary, and where other pro-fibrotic pathways, driven by metabolic comorbidities like severe insulin resistance, become dominant and are not adequately addressed by ASK1 inhibition alone (Powell et al., 2021; Peng et al., 2024; Zhu and Cai, 2025).

As another emerging target, Bruton’s tyrosine kinase (BTK) is a critical signaling molecule within the B-cell receptor and other innate immune pathways, with its inhibition now being explored for modulating macrophage function in liver disease. Mechanistically, BTK inhibition can block pro-inflammatory signaling downstream of Fc receptors and certain TLRs, reducing the production of mediators like TNF-α and IL-1β (Palumbo et al., 2017; Purvis et al., 2020). Intriguingly, BTK inhibition may also promote a shift towards M2 polarization. This potential dual action—suppressing M1-like inflammation while simultaneously promoting an M2-like reparative state—makes BTK a novel and attractive therapeutic target. Supporting this concept, preclinical studies using BTK inhibitors have shown reduced monocyte/macrophage recruitment and activation in the liver, leading to improved metabolic inflammation (Purvis et al., 2020).

## 4 Differential responses of LRM subsets to immunomodulation in specific CLDs

### 4.1 MASH

MASH is characterized by a profound remodeling of the LRM landscape. The prevailing lipotoxic environment drives resident KCs towards a senescent, pro-inflammatory state, which progressively erodes their homeostatic capacity (Ma et al., 2024). Concurrently, a robust upregulation of the CCL2-CCR2 axis prompts a massive influx of pro-inflammatory Ly-6C^hi^ MoMφs, which subsequently emerge as the numerically and functionally dominant macrophage population (Ganguly et al., 2024). This MoMφ dominance is a key driver of inflammation, hepatocyte death, and fibrogenesis in MASH.

PPAR agonists exert their beneficial effects by concurrently targeting both the metabolic and inflammatory dysregulation inherent to MASH. It is understood that they preferentially act on KCs to promote a switch towards an anti-inflammatory M2 phenotype, helping to suppress the chronic low-grade inflammation (Shi Q. et al., 2025). It is further hypothesized that pleiotropic agents like the pan-PPAR agonist lanifibranor may have broader effects, potentially influencing the phenotype of both resident KCs and infiltrating MoMφs, a mechanism that could account for its observed efficacy in reducing both inflammation and fibrosis (Ma et al., 2024; Yu et al., 2025).

In contrast, CCR2/CCR5 antagonists like cenicriviroc were designed with a more singular focus: to block the recruitment of the pathogenic Ly-6C^hi^ MoMφ population. The therapeutic hypothesis was that by stemming this influx, the primary drivers of inflammation and fibrosis would be intercepted before entering the liver. The clinical failure of this strategy in Phase III, however, prompts a re-evaluation of this premise. It suggests that in patients with established advanced fibrosis, merely blocking the recruitment of new MoMφs may be “too little, too late”. At this stage, the pathology is likely perpetuated by self-sustaining pathogenic feedback loops involving macrophage populations already established within the liver, such as SAMs, which engage in persistent pro-fibrotic crosstalk with HSCs through autocrine and paracrine signaling. This entrenchment of disease mechanisms makes the fibrotic process less dependent on the continuous influx of peripheral monocytes. This reality, combined with the possibilities that redundant pro-fibrotic pathways have become dominant or that patient heterogeneity diluted the therapeutic effect, provides a more comprehensive explanation for the trial’s outcome.

### 4.2 ALD

In ALD, the LRM landscape is profoundly altered by the confluence of direct ethanol-induced toxicity and increased gut permeability. A critical consequence of this compromised gut barrier is the translocation of bacterial LPS to the liver, where it potently engages TLR4 on KCs (Mak and Shekhar, 2025). This engagement triggers robust KC activation and the production of pro-inflammatory cytokines, creating an intense inflammatory milieu that drives hepatocyte injury and the subsequent recruitment of large numbers of Ly-6C^hi^ MoMφs, thereby establishing a self-amplifying inflammatory loop that perpetuates tissue damage (Slevin et al., 2020).

Given the central pathogenic role of the LPS-TLR4 axis in ALD, its inhibition represents a highly rational therapeutic strategy. The therapeutic premise is that by blocking TLR4 on KCs, these agents can intercept the primary inflammatory trigger in ALD (Mou et al., 2022). This intervention is predicted to reduce the secretion of key cytokines like TNF-α, which in turn would diminish the chemotactic signals responsible for the recruitment of pathogenic MoMφs, ultimately ameliorating liver injury (Tang et al., 2025).

Similar to MASH, the intense inflammatory environment in ALD drives the CCL2-dependent recruitment of Ly-6C^hi^ MoMφs. Therefore, targeting this axis by blocking MoMφ infiltration with CCR2/CCR5 antagonists represents another compelling therapeutic strategy (Madan et al., 2024). The intended outcome of this approach is to mitigate the acute liver injury characteristic of severe ALD by limiting the number of these potent inflammatory effector cells.

### 4.3 Viral hepatitis and liver fibrosis/cirrhosis

In chronic viral hepatitis, LRMs assume a paradoxical role, functioning as both antiviral effectors and facilitators of viral persistence (Simón-Codina et al., 2024). While they are integral to the initial antiviral response, they can be co-opted by viruses to establish a state of immune tolerance that supports chronic infection (Chi et al., 2023). This persistent, low-grade LRM activation fosters a microenvironment of sustained inflammation, which serves as a key catalyst for immune-mediated hepatocyte damage and the inexorable progression to fibrosis (Wang J. et al., 2024b).

Navigating therapeutic immunomodulation in viral hepatitis presents a formidable clinical challenge. While strategies aimed at augmenting antiviral immunity (e.g., with checkpoint inhibitors) hold the potential for viral clearance, they carry the inherent risk of exacerbating immune-mediated liver injury (Biehl et al., 2021). Conversely, conventional immunosuppression, while capable of dampening inflammation, does so at the cost of permitting unchecked viral replication. In this context, macrophage-targeted therapies emerge as a potentially more refined strategy, offering a pathway to selectively quell pathological inflammation without globally compromising the adaptive immune responses essential for viral containment (Qian et al., 2024).

In the context of established fibrosis, irrespective of the underlying etiology, macrophages assume a pivotal role in dictating the potential for disease resolution. Consequently, the therapeutic paradigm shifts from mere inflammation control to the active promotion of a pro-resolving macrophage phenotype, typified by Ly-6C^lo^ MoMφs and reparative SAMs. These cells are the primary source of matrix-degrading metalloproteinases (MMPs) required for the enzymatic dismantling of the fibrotic scar (Fallowfield et al., 2007). Furthermore, they can induce apoptosis of activated HSCs, thus eliminating the primary cellular source of collagen production. Therapeutic strategies that can orchestrate this phenotypic switch in macrophages hold immense promise for inducing the regression of advanced liver fibrosis and cirrhosis.

## 5 Clinical implications and future directions

### 5.1 Challenges in translating preclinical findings to clinical practice

A fundamental challenge impeding CLD drug development is the vast heterogeneity inherent to the patient population. While preclinical models frequently rely on genetically homogenous animals subjected to a uniform injurious stimulus, human CLD is a multifactorial syndrome, with a trajectory shaped by a complex interplay of genetics, diet, comorbidities, and gut microbiome composition. For example, genetic variants such as PNPLA3 and TM6SF2 are potent drivers of MASH progression and may create a disease state less amenable to therapies targeting purely inflammatory pathways (Kozlitina et al., 2014; Lavrado et al., 2024). Similarly, comorbidities like type 2 diabetes induce a more aggressive, fibrosis-prone MASH phenotype, potentially requiring more potent or combination therapies (Cai et al., 2025; Younossi et al., 2025). Consequently, a therapeutic strategy targeting a single pathway is unlikely to be universally effective, and an intervention beneficial for early-stage inflammation may be futile against the deeply entrenched pathological feedback loops of advanced cirrhosis. This disconnect between preclinical models and clinical reality likely contributed to the failure of mechanistically focused agents like selonsertib and cenicriviroc when tested in broad, unselected Phase III populations (Saldarriaga et al., 2024).

Addressing this heterogeneity necessitates the development of predictive biomarkers capable of identifying patients most likely to respond to a given therapy. Such tools are essential for enabling patient stratification, which would not only improve the likelihood of clinical trial success but also allow for more efficient, targeted study designs. The ongoing revolution in 'omics’ technologies offers a promising path forward, facilitating the identification of molecular and cellular signatures linked to disease progression. For instance, quantifying signatures of specific LRM subsets (e.g., circulating soluble TREM2 as a surrogate marker for LAM activity) or identifying specific gut microbiome profiles associated with pro-inflammatory LRM activation could 1 day be used to enrich clinical trials with patients most likely to benefit from therapies targeting those distinct populations (Hendrikx et al., 2022; Liebold et al., 2023).

Beyond efficacy, the deliberate modulation of the immune system carries inherent safety considerations. For example, a therapy that blocks inflammatory monocyte recruitment could compromise host defense, increasing susceptibility to infection. Similarly, therapeutically skewing macrophages toward an M2-like phenotype could, in theory, blunt anti-tumor surveillance, a critical concern in patients with cirrhosis who are at high risk for hepatocellular carcinoma (HCC). Therefore, a deep mechanistic understanding of any immunomodulatory agent, coupled with vigilant patient monitoring, is paramount to navigating the fine line between therapeutic benefit and immune-related adverse events.

### 5.2 The LRM-HCC axis and implications for immunotherapy

The progression from CLD to HCC represents the most feared outcome, and LRMs are pivotal players in this transition. The chronic inflammatory microenvironment orchestrated by pro-inflammatory KCs and MoMφs creates a mutagenic milieu that promotes hepatocyte transformation (Ma et al., 2024; Wu et al., 2025). As a tumor develops, the LRM landscape undergoes a further dramatic shift. LRMs are recruited to the tumor mass, where the local microenvironment, rich in factors like IL-10 and TGF-β, reprograms them into tumor-associated macrophages (TAMs) (Yang and Zhang, 2017).

These TAMs predominantly adopt an M2-like, immunosuppressive phenotype. They actively suppress anti-tumor immunity through multiple mechanisms: they release cytokines that inhibit T-cell proliferation and function, remodel the ECM to facilitate tumor invasion, and promote angiogenesis to support tumor growth (Yang and Zhang, 2017; Zheng et al., 2023). Crucially, TAMs are a major source of immune checkpoint ligands, particularly PD-L1 (Li Z. et al., 2024). The expression of PD-L1 on TAMs allows them to directly engage the PD-1 receptor on cytotoxic T-lymphocytes, inducing their exhaustion and anergy, thereby creating a potent shield that protects the tumor from immune-mediated destruction (Wang L. et al., 2024c; Wang et al., 2024a).

This understanding has profound implications for HCC therapy, particularly for immune checkpoint inhibitors (ICIs) like anti-PD-1/PD-L1 antibodies. The efficacy of ICIs is often limited by the immunosuppressive tumor microenvironment, in which TAMs are a key component. This provides a strong rationale for combination therapies. Strategies that deplete or repolarize TAMs—for instance, by inhibiting the CSF1R pathway—could dismantle the immunosuppressive shield and synergize with ICIs to unleash a more effective anti-tumor T-cell response (Zhu et al., 2019). Therefore, therapies originally developed for fibrosis that can modulate LRM phenotype may find new life as adjuncts to immunotherapy in HCC, strengthening the translational impact of LRM-targeted research.

### 5.3 Future research priorities

A critical research priority is the systematic application of scRNA-seq and other single-cell technologies to human liver tissue (Ramachandran et al., 2019; Xiong et al., 2019). These technologies provide the high-fidelity resolution needed to map LRM heterogeneity directly within human patients across the spectrum of CLDs (Wang et al., 2023; Li B. et al., 2024). Such efforts are essential to 1) robustly validate or refute findings from preclinical models, 2) identify novel, human-specific macrophage subsets, and 3) uncover disease-specific LRM signatures that can be leveraged for biomarker development and as novel therapeutic targets (Fabre et al., 2023).

Intensified investigation into the gut-liver axis represents another key research frontier. The continuous trafficking of microbial products and metabolites from the gut is now understood to be a primary determinant of the activation state of KCs and other LRMs (Miyamoto et al., 2024). Establishing a mechanistic link between specific dysbiotic signatures or microbial metabolite profiles and the resultant LRM landscape in diseases like MASH and ALD is therefore a high priority. For instance, specific microbial metabolites such as short-chain fatty acids (SCFAs) can directly modulate KC function through receptors like G protein-coupled receptor 43 (GPR43), while secondary bile acids are known to signal through the Takeda G protein-coupled receptor 5 (TGR5) on macrophages, influencing their inflammatory tone (Tan et al., 2023; Haag et al., 2025). This knowledge could pave the way for novel therapeutic strategies, such as engineered probiotics or targeted dietary interventions, designed to modulate LRM function via the gut.

Finally, the multifactorial pathogenesis of CLDs strongly suggests that combination therapies will be required to achieve maximal therapeutic efficacy. Future strategies must therefore focus on the rational design of regimens that combine agents with complementary mechanisms. For example, a logically designed combination could pair a CCR2 antagonist, to block the recruitment of new inflammatory monocytes, with a PPAR agonist, to reprogram macrophages already resident within the liver towards a reparative phenotype. Such a dual-pronged strategy, simultaneously targeting both the influx and the function of pathogenic macrophages, could produce synergistic therapeutic effects and represents the next logical step in the evolution of LRM-targeted therapies.

## 6 Conclusion

The study of LRMs is central to the ongoing paradigm shift in hepatology, steering the field toward an era of targeted immunomodulation and precision medicine. The recognition that LRMs are a heterogeneous consortium with distinct, and often opposing, functions has fundamentally reshaped our understanding of CLD pathogenesis. This refined conceptual framework has unveiled a landscape of novel therapeutic targets aimed at selectively modulating specific LRM subsets. However, translating these concepts into clinical success has been fraught with challenges. The failures of recent, mechanistically targeted agents serve as a crucial reminder of the immense hurdles posed by patient heterogeneity and the recalcitrance of advanced disease. The critical role of these cells in shaping the tumor microenvironment further extends their therapeutic relevance into the realm of immuno-oncology. The future of CLD therapy will therefore depend on a deeper and more granular integration of LRM biology into therapeutic design. By harnessing the power of single-cell technologies, developing predictive biomarkers for patient stratification, and deploying rationally designed combination therapies, the field is poised to finally move beyond the “one-size-fits-all” paradigm. The ultimate ambition is to precisely tailor treatments to the unique macrophage-driven pathology of each individual, ushering in an era where halting or even reversing the course of CLD becomes a clinical reality.
